# Gastrodin Ameliorates Type II Diabetes Through the YY1–FXR–Bile Acid Axis

**DOI:** 10.3390/ijms27104593

**Published:** 2026-05-20

**Authors:** Xiaolin Zhang, Yushan Du, Penghui Yang, Shiji Li, Fengya Cui, Xinran Li, Xinyue Du, Bingyao Sun, Yulu Ma, Wenjie Sui, Min Zhang, Jing Meng

**Affiliations:** 1 State Key Laboratory of Food Nutrition and Safety, College of Food Science and Engineering, Tianjin University of Science and Technology, No. 29, 13th Avenue, TEDA, 300457 Tianjin, China; 2 Tianjin Agricultural University Nutritious and Healthy Food Sino-Thailand Joint Research Center, Tianjin Agricultural University, No. 22, Jinjing Road, 300380 Tianjin, China; 3 Guangxi Key Laboratory of Early Prevention and Treatment for Regional High Frequency Tumor, Key Laboratory of Early Prevention and Treatment for Regional High Frequency Tumor, Ministry of Education, Guangxi Medical University, No. 22, Shuangyong Road, 530021 Nanning, China

**Keywords:** type II diabetes mellitus, gastrodin, gut microbiota, bile acid metabolism, YY1, FXR

## Abstract

Type II diabetes mellitus (T2DM), a chronic metabolic disorder characterized by insulin resistance, is often accompanied by dysregulated bile acid metabolism. Although gastrodin, a bioactive compound derived from Gastrodia elata, has demonstrated potential in diabetes management, its therapeutic mechanisms remain incompletely understood. The aim of this study is to investigate the therapeutic effects and potential mechanisms of gastrodin on T2DM mice from the perspective of bile acid metabolism. In this study, we found that gastrodin could not only reduce lipid accumulation, reduce inflammation, improve antioxidant capacity, alleviate oxidative stress, change the composition of intestinal flora, and improve the disorder of flora caused by the disease in T2DM mice, but also target Yin yang 1 (YY1) to reduce the expression level of YY1 in the liver under a high-fat diet condition. At the same time, YY1 negatively regulates the expression level of Farnesoid X Receptor (FXR), which increases the expression level of FXR, inhibits the enzyme activity of Cholesterol-7α-hydroxylase (CYP7A1) through Small Heterodimer Partner (SHP), reduces the production of chenodeoxycholic acid (CDCA) in the liver, and further affects the production of secondary bile acids through liver–intestinal circulation, promoting the secretion of Glucagon-Like Peptide-1 (GLP-1) and insulin, thereby reducing blood glucose. At the same time, combined with the results of HE staining, gastrodin can reduce the pathological damage of the liver and pancreas in type II diabetic mice, repairing their normal morphology and function. It provides a direct pathological basis for the improvement of diabetes and liver complications, provides theoretical support for the subsequent research and development of precision targeted drugs, provides experimental basis for the development of new natural hypoglycemic drugs, and promotes the transformation and application of the modernization of traditional Chinese medicine in the field of metabolic diseases.

## 1. Introduction

Type II diabetes mellitus (T2DM) is a severe chronic metabolic disease. Its main pathophysiological mechanism is progressive failure of pancreatic-β-cell function, leading to insufficient insulin secretion and insulin resistance [1]. Type II diabetes is the main type of diabetes in our country, accounting for more than 90% of the total number of diabetic patients [2]. At present, the commonly used oral hypoglycemic drugs on the market are metformin or sulfonylureas. However, long-term use of such drugs can easily foster dependence, and unauthorized withdrawal of the drugs will aggravate insulin resistance [3]. Therefore, the need to find a new strategy for the treatment of type II diabetes is urgent.

More and more studies have proven that the relationship between human health and gut microbiota is bidirectional, dynamic and highly individualized [4]. The imbalance of gut microbiota is inextricably linked to more than 80% of chronic diseases. There is a close and complex bidirectional relationship between gut microbiota and diabetes, especially type II diabetes [5,6]. Diabetes itself can also lead to a disorder of intestinal flora. The state of hyperglycemia can change the intestinal environment and selectively promote the growth of certain pathogenic bacteria.

Bile acid (BA) is a kind of steroid compound synthesized in the liver, with cholesterol as the precursor. The liver is the only organ for bile acid synthesis. In the liver, cholesterol is used as the substrate and is decomposed in two ways. Cholesterol in the liver is decomposed into chenodeoxycholic acid (CDCA) by cholesterol-7α-hydroxylase (CYP7A1), or into cholic acid (CA) by the 12α-hydroxyl group introduced by sterol-12α-hydroxylase (CYP8B1) [7,8]. After these compounds are synthesized in the liver, they are mostly conjugated with glycine or taurine to form conjugated bile acids to enhance their water solubility and physiological activity. About 90% of the bile acids in the intestine are reabsorbed in the terminal ileum through an active transport pathway mediated by apical sodium-dependent bile acid transporter (ASBT) and returned to the liver through the portal vein system [9]. Thus, primary bile acids complete enterohepatic circulation, enabling bile acid reuse. The small amount of bile acids not reabsorbed are metabolized by gut microbiota in the colon into free bile acids, which are subsequently converted into secondary bile acids through reactions such as 7α-dehydroxylation. These secondary bile acids re-enter enterohepatic circulation; only a minimal amount of hydrophobic secondary bile acids cannot be reabsorbed and are ultimately excreted in feces [10].

As a core member of the bile acid nuclear receptor family, FXR (farnesoid X receptor) is a key regulatory molecule connecting bile acid metabolism and glucose metabolism homeostasis [11,12]. Chenodeoxycholic acid (CDCA) is the most potent activator of FXR, followed by lithocholic acid (LCA) and deoxycholic acid (DCA) [13]. Studies have shown that the activation of FXR can directly regulate the insulin secretion function of islet β cells, and FXR can also indirectly improve insulin resistance by regulating lipid metabolism homeostasis, suggesting that FXR is a potential important target for the treatment of diabetes.

YY1 (Yin Yang 1) protein is a zinc finger transcription factor belonging to the GLI-Kruppel family. Its coding gene is located on human chromosome 14, widely expressed in various tissues and cells of the body, and mainly locates in the nucleus to perform transcriptional regulation [14]. It is a key multifunctional molecule involved in gene expression regulation, cell physiological process regulation and disease occurrence and development [15]. Previous studies have confirmed that YY1, as a multifunctional transcription factor, negatively regulates the transcription of FXR, the nuclear bile acid receptor, and indirectly interferes with the homeostasis of bile acid metabolic pathway, thereby affecting the balance of glucose and lipid metabolism [16]. However, studies on the interaction between bile acids and YY1 are still focused on metabolic diseases, and the integrity of the regulatory network has not been fully elucidated. The YY1–FXR axis is a key molecular pathway connecting bile acid metabolism with glucose and lipid metabolism, suggesting that this axis may be a potential target for clinical intervention of metabolic diseases.

**Figure 1:**
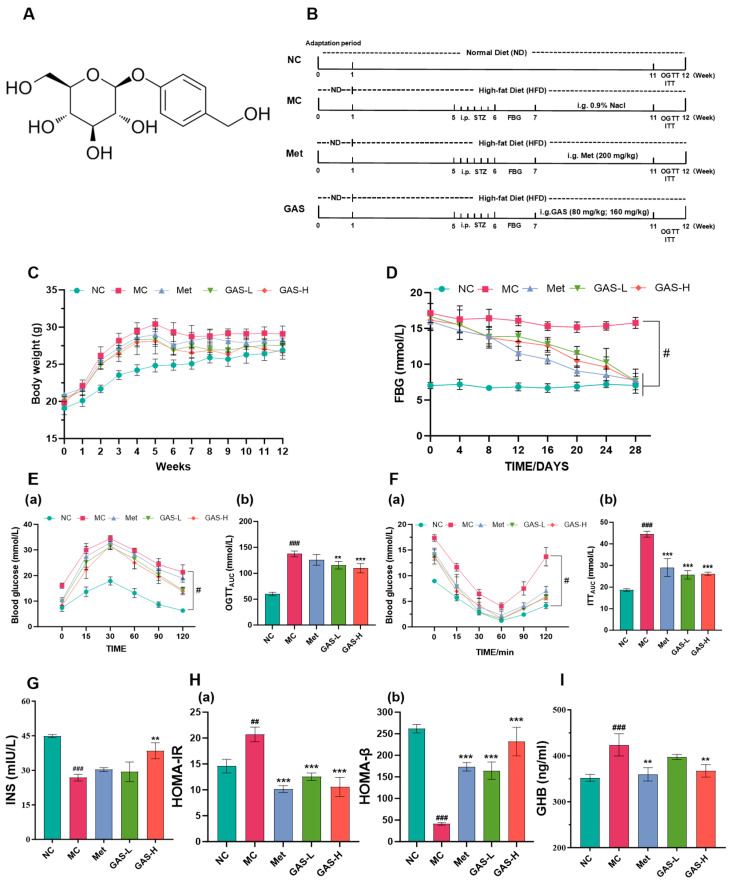
The effect of GAS on blood glucose in T2DM mice. (**A**) The chemical formula of gastrodin; (**B**) Animal experiment procedure; (**C**) Body weight; (**D**) Fasting blood glucose; (**E**) (**a**) OGTT; (**b**) OGTT_AUG_; (**F**) (**a**) ITT (**b**) ITT_AUG_; (**G**) INS; (**H**) (**a**) HOMA-IR (**b**) HOMA-β; (**I**) GHB. Statistical significance: # *p* < 0.05, ## *p* < 0.01, ### *p* < 0.001, MC group vs. NC group; ** *p* < 0.01, *** *p* < 0.001, Treatment groups vs. MC group.

Gastrodia elata Blume is a precious traditional Chinese herb with medicine–food homology, possessing diverse pharmacological effects and clinical applications [17]. Gastrodin (GAS, C_13_H_18_O_7,_ Figure 1A), the principal bioactive constituent of G. elata, is a phenolic glycoside chemically known as 4-hydroxybenzyl alcohol 4-O-β-D-glucopyranoside. It exhibits poor liposolubility but high water solubility, has an oral bioavailability of approximately 30%, and is capable of crossing the blood–brain barrier [18].

Gastrodin is commonly employed in the field of neuroprotection, exhibiting sedative and antiepileptic effects. However, research on gastrodin has become increasingly in-depth in recent years; it has emerged as a significant therapeutic candidate for disorders of carbohydrate and lipid metabolism. Studies on gastrodin’s role in regulating bile acid metabolism have been progressively advanced.

At the signaling pathway level, gastrodin can activate the nuclear receptor FXR signaling pathway to alleviate the depression-like behavior of diabetic mice caused by chronic stress in the mouse model of diabetic depression [19]. Gastrodin can also directly activate peroxisome-proliferator-activated receptor (PPAR)α, enhance hepatic fatty acid β-oxidation, reduce intrahepatic triglyceride deposition, decrease the substrate supply for bile acid synthesis, and synergistically improve bile acid metabolic abnormalities associated with Nonalcoholic steatohepatitis (NASH) [20]. Furthermore, the inhibitory effect of gastrodin on the NF-κB inflammatory signaling pathway reduces the interference of inflammatory factors (TNF-α, IL-6) on the expression of bile acid transporters, thereby further maintaining bile acid metabolic homeostasis and creating a synergistic protective effect of “metabolic regulation–inflammatory suppression–bile acid homeostasis”.

In recent years, with advancements in metabolic disease research, the role and mechanisms of gastrodin in regulating glucose metabolic homeostasis have emerged as a focal point of investigation. Several studies have demonstrated that gastrodin promotes the phosphorylation of GATA-binding proteins via the PI3K/AKT signaling pathway, thereby enhancing the expression and activity of insulin receptors on cell surfaces [21]. It also modulates glucose metabolism through multiple pathways, including reducing hepatic glucose output by activating the AMPK/Nrf2 signaling pathway [22]. However, the binding mechanisms of gastrodin to receptors such as YY1 and FXR in regulating bile acid metabolism and consequently glucose metabolism via gut microbiota remain incompletely elucidated and require further validation. Based on this, our study aims to elucidate the regulatory effects of gastrodin on glucose metabolic homeostasis through experimental approaches, revealing its comprehensive mechanism by targeting the YY1–FXR axis to modulate gut microbiota–bile acid metabolism for maintaining glucose metabolic homeostasis. This work provides a scientific foundation and experimental basis for developing gastrodin-related natural hypoglycemic agents and exploring novel targeted intervention strategies for metabolic diseases.

## 2. Results

### 2.1. Effects of GAS on Glucose Metabolism and Insulin Sensitivity in HFD/STZ Diabetic Mice

Type II diabetes is mainly caused by an unhealthy lifestyle, including a diet high in fat and sugar [19]. After a week of adaptation, the experimental group began to carry out a diabetes high-fat diet for about four weeks. When the body weight reached about 30 g, the HFD group began to establish the diabetes model by injection of Streptozotocin (STZ 50 mg/kg, five consecutive days). The control group continued to eat the normal diet. After the model was successfully established, the HFD group was divided into 4 equal groups (*n* = 6) and given Met and GAS by gavage (Figure 1B). During the experiment, the body weight of the mice in the control group continued to increase steadily, while the body weight of the mice in the model group increased significantly, and there was a significant difference in body weight between the mice in the model group and the NC group at the fifth week (*p* < 0.01). At the same time, the body weight of the mice in the MC group was maintained at a high level throughout the experiment. At the end of the drug intervention, there was no significant difference in the body weight of the treated mice compared with the normal mice at *p* < 0.05, which was close to the normal mice (Figure 1C). These results suggest that GAS can improve living status and weight gain in STZ-induced type II diabetic mice.

High fat and high glucose can lead to obesity and insulin resistance in mice, but it is not sufficient to establish a typical type 2 diabetes model. Therefore, low-dose STZ injections were administered starting from week five. Fasting blood glucose > 11.1 mmol/L was defined as a diabetic mouse model. After successful modeling, GAS was given by gavage. At the beginning of the administration, the fasting blood glucose of the diabetic mice was approximately 17 mmol/L. After administration, the blood glucose of each group decreased to 11.1 mmol/L and below, the blood glucose was stable, and no rebound of blood glucose was observed in a short time. On day 28 of administration, compared with the NC group, the blood glucose of the MC group increased significantly (*p* < 0.05). Compared with the model group, the blood glucose of the gastrodin treatment group was significantly decreased (*p* < 0.05). In addition, there was no significant difference in the glucose-lowering effect between the gastrodin and metformin groups (Figure 1D). These results suggest that drug intervention with GAS reduces fasting blood glucose values in T2DM mice.

The oral glucose tolerance test (OGTT) assesses the function of islet β cells and the body’s ability to regulate blood glucose [23]. After gavage of glucose, the blood glucose level of each group reached the highest value at 30 min, and at each time point, the blood glucose level of the MC group was higher than that of the other groups (*p* < 0.05). The area under the curve decreased during the entire OGTT in the treated group compared with the MC group (*p* < 0.05), indicating that the level of insulin resistance was relieved in diabetic mice after GAS treatment (Figure 1E). The insulin tolerance test (ITT) can directly reflect the sensitivity of mice to insulin. After insulin injection, the blood glucose level of each group decreased to the lowest level at 60 min. Compared with the control group, the blood glucose of the model group decreased slowly, and the blood glucose of each time point was higher than that of the other groups (*p* < 0.05). Compared with the model group, the area under the curve was significantly reduced in the administration group during the whole ITT period (*p* < 0.05), indicating that after the administration of GAS, the sensitivity of diabetic mice to insulin can be improved and insulin resistance can be alleviated (Figure 1F).

Insulin (INS) is a polypeptide hormone secreted by pancreatic β cells. Its main function is to regulate blood glucose level and promote the uptake, utilization and storage of glucose. The functional status of islet β cells can be assessed by detecting insulin levels in mice [23]. Compared with the NC group, the insulin level in the MC group was significantly decreased (*p* < 0.001), and the blood glucose value was correspondingly increased, indicating that the islet β cells in the model group were damaged and insulin secretion was insufficient. After administration, compared with the MC group, there was no significant difference in insulin level between the Met group and the model group, indicating that metformin could not repair islet β cells and promote insulin secretion. However, compared with the MC and Met groups, the insulin level in the GAS-H group shows a significant upward trend (*p* < 0.05) (Figure 1G). The HOMA-IR and HOMA-β indexes showed that GAS could improve insulin resistance and the function of islet β cells in mice (Figure 1H).

The formation of glycosylated hemoglobin (GHB) is a slow, continuous process of non-enzymatic glycation. The rate of GHB formation was positively correlated with blood glucose concentration [24]. Compared with the NC group, the GHB level in the MC group was significantly higher than that in the other groups (*p* < 0.001); after GAS drug intervention treatment, the GHB level decreased significantly (*p* < 0.01). GAS can reduce GHB content and blood glucose concentration. All these results suggest that GAS can improve hyperglycemia induced by HFD/STZ, alleviate insulin resistance in T2DM mice, and may play an important role in the repair of islet β cells (Figure 1I).

### 2.2. The Effects of GAS on Physiological and Biochemical Parameters in HFD/STZ Diabetic Mice

**Figure 2:**
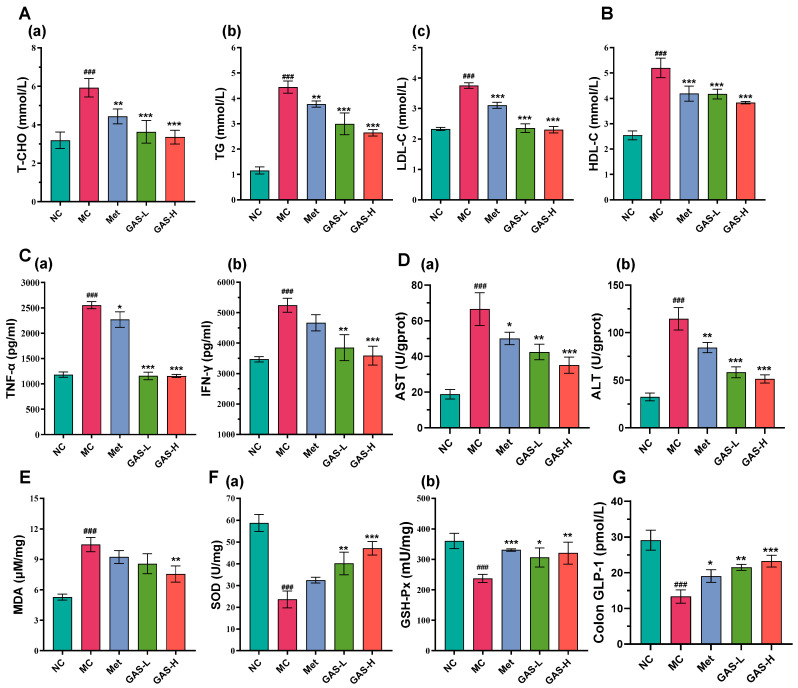
The effects of GAS on physiological and biochemical parameters in T2DM mice. (**A**) (**a**) T-CHO, (**b**) TG and (**c**) LDL-C; (**B**) HDL-C; (**C**) (**a**) TNF-α and (**b**) IFN-γ; (**D**) (**a**) AST and (**b**) ALT; (**E**) MDA; (**F**) (**a**) SOD and (**b**) GSH-Px; (**G**) Colon GLP-1. Statistical significance: ### *p* < 0.001, MC group vs. NC group; * *p* < 0.05, ** *p* < 0.01, *** *p* < 0.001, Treatment groups vs. MC group.

Four items of blood lipids are important indicators used to detect blood lipid levels. Among them, the increase in Total cholesterol (TC), Triglyceride (TG) and Low density lipoprotein cholesterol (LDL-C) is an important risk factor for cardiovascular diseases such as coronary heart disease and atherosclerosis [25]. In this study, compared with the NC group, the TC, TG, and LDL-C levels in the experimental group showed higher levels due to the high-fat diet, the levels in the MC group increased significantly (*p* < 0.001), and the TC, TG, and LDL-C levels decreased after GAS drug treatment (*p* < 0.01) (Figure 2A). HDL-C levels are inversely associated with the risk of atherosclerotic cardiovascular disease [26]. However, in T2DM mice, due to the high-fat diet-induced diabetes, the intake and metabolism of lipids are increased. At this time, the liver will increase the synthesis and secretion of High density lipoprotein cholesterol (HDL-C) to help transport excessive TG, resulting in the increase in HDL-C level. The high-fat diet resulted in higher HDL levels in the experimental group than in the NC group (*p* < 0.001). After administration of GAS, HDL levels showed a dose-dependent downward trend compared with the MC group (*p* < 0.001) (Figure 2B). These results suggest that intervention with GAS can alleviate lipid metabolism disorders caused by insulin resistance in diabetic mice.

In the diabetic model, Tumor necrosis factor-α (TNF-α) and Interferon-γ (IFN-γ) are important proinflammatory factors, which can induce the production of other inflammatory factors and further amplify the inflammatory response [27]. Compared with the NC group, the levels of inflammatory factors in the serum of the mice in each experimental group were higher due to diabetes (*p* < 0.001). Compared with the MC group, the level of inflammation in the GAS group decreased (*p* < 0.01), indicating that GAS drug intervention can effectively alleviate the inflammatory response (Figure 2C).

The ratio of aspartate aminotransferase to alanine aminotransferase is commonly used to assess the presence of liver tissue lesions. Compared with the NC group, the Aspartate aminotransferase (AST) and Alanine aminotransferase (ALT) content in the MC group showed a significant increasing trend (*p* < 0.001). It is speculated that under the disease state, after high-fat diet feeding and STZ injection, the mice not only showed increased blood glucose, but also showed a certain degree of liver loss. However, after GAS drug intervention, the AST and ALT content showed a significant downward trend compared with MC group (*p* < 0.01), which proves that GAS could alleviate the phenomenon of liver injury in mice under disease conditions (Figure 2D).

Increased Malondialdehyde (MDA) levels usually indicate increased oxidative stress, which may lead to cell damage and the occurrence of a variety of diseases. Compared with the NC group, the MDA level in the MC group was the highest (*p* < 0.001), indicating that diabetes caused oxidative stress in the organism. Compared with the MC group, the MDA level after GAS administration showed a downward trend in a dose-dependent manner (*p* < 0.05) (Figure 2E). Superoxide dismutase (SOD) and glutathione peroxidase (GSH-px) are important antioxidant enzymes in the body. Due to the disease response, SOD and GSH-px levels were significantly lower in the MC group than in the NC group (*p* < 0.001), and the activity levels of both enzymes were significantly increased after GAS administration (*p* < 0.05). This indicates that GAS can alleviate oxidative stress in diabetic mice, reduce cell damage and avoid the occurrence of a variety of diseases (Figure 2F).

These results suggest that GAS can regulate lipid metabolism disorders, reduce inflammation and alleviate oxidative stress in T2DM mice.

Glucagon-like peptide-1 (GLP-1) is an incretin hormone secreted by intestinal L cells and plays a key role in blood glucose regulation and energy metabolism [28]. Compared with the NC group, the content of GLP-1 in the colon tissue of the MC group was significantly decreased (*p* < 0.001), indicating that the disease state led to intestinal L cell damage and decreased secretory function of the L cells. After the administration of GAS, the GLP-1 content was significantly increased (*p* < 0.05), indicating that GAS drug treatment can increase the secretion of GLP-1 in the intestine of mice and reduce the damage of intestinal L cells (Figure 2G).

### 2.3. The Effects of GAS on Gut Microbial Diversity in HFD/STZ Diabetic Mice

**Figure 3:**
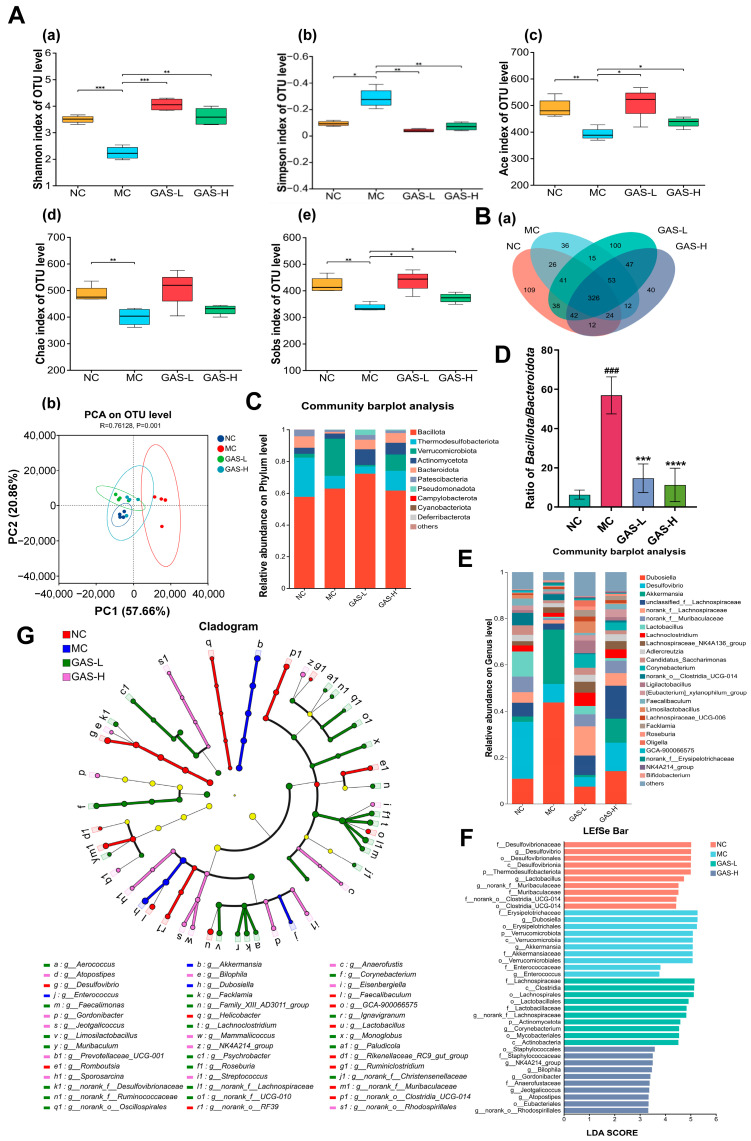
GAS regulates the composition and function of the intestinal microbiota in T2DM mice. (**A**) Shannon index, Simpson index, Ace index, Chao index and Sobs index; (**B**) (**a**) Venn diagram (**b**) PCA analysis at the OTU level; (**C**) Bar chart of the community at the phylum level; (**D**) Relative abundance of *Bacillota/Bacteroidota*; (**E**) Bar chart of the community at the genus level; (**F**) LEfSe classification tree analysis of key genera; (**G**) LDA score plot of bacterial taxa with an LDA score > 2. Statistical significance: * *p* < 0.05, ** *p* < 0.01, *** *p* < 0.001, **** *p* < 0.0001.

In α diversity analysis of intestinal flora, the Simpson index and the Shannon index are commonly used to evaluate the core index of microbial community diversity, and the Ace index, the Chao (Chao1) index and the Sobs index are used to evaluate the species richness of the microbial community [29]. Compared with the NC group, the Simpson index was increased and the Shannon index was decreased in the MC group, while GAS significantly decreased the Simpson index and increased the Shannon index, thereby increasing the diversity of the gut microbiota. Compared with the NC group, the Ace, Chao and Sobs indexes of the MC group significantly decreased, and the ACE, Chao and Sobs indexes of the T2DM mice treated with GAS increased, indicating that GAS could promote the richness of intestinal microbiota (Figure 3A).

The 16s high-throughput sequencing data showed that the number of OTUs in NC, MC, GAS-L, and GAS-H groups was 618, 533, 662, and 556, respectively. The total number of OTUs across all groups was 326. There were 109 unique bacteria in the NC group, 36 in the MC group, 100 in the GAS-L group, and 40 in the GAS-L group. This suggests that the gut microbiota recovered somewhat after GAS treatment. Next, principal component analysis (PCA) was used to further investigate the β-diversity of gut microbiota in the three groups. PCA results clearly showed that there were significant differences in gut microbiome composition between the NC group and the MC group, while GAS-L and GAS-H groups were more similar to the NC group (Figure 3B).

Compared with the NC group, the abundance of *Bacillota* and *Verrucomicrobiota* in the MC group increased to varying degrees, and the relative abundance was significantly reduced after GAS treatment intervention. The relative abundance of *Thermodesulfobacteriota*, *Actinomycetota* and *Bacteroidota* decreased, and increased significantly after GAS treatment. At the same time, in type II diabetes, an increased ratio of *Firmicutes* to *Bacteroidetes* implies an increased burden of glucose metabolism, which is associated with insulin resistance in the context of a high-energy diet [30]. Compared with the NC group, the ratio of *Bacillota*/*Bacteroidota* was significantly increased in the MC group, which was restored after GAS intervention (Figure 3C,D).

In addition, the data showed that the dominant gut microbiota at the genus level included *Dubosiella* and *Desulfovibrio*, this was followed by *Akkermansia, unclassified_f__Lachnospiraceae, norank_f__Lachnospiraceae* and *norank_f__Muribaculaceae*. Compared with NC, the intestinal microbiota in MC showed an increase in the abundance of *Dubosiella* and *Akkermansia* at the genus level. The abundance of *Desulfovibrio*, *unclassified_f__Lachnospiraceae*, *norank_f__Lachnospiraceae*, *norank_f__Muribaculaceae*, *Lactobacillus*, and *Lachnoclostridium* decreased, while GAS treatment restored the relative abundance at the genus level. These results indicated that the STZ + HFD induced T2DM model could interfere with intestinal microbiota in mice, while GAS changed the composition of intestinal microbiota in T2DM mice to a certain extent (Figure 3E).

LEfSe’s multi-level species system was used to analyze the diversity of gut microbiota, and the results showed that the relative abundance of 45 species differed among the four groups, of which 11, 3, 19 and 12 species belonged to NC, MC, GAS-L and GAS-H, respectively. The relative abundance of *Desulfovibrionaceae, Thermodesulfobacteriota* and *Lactobacillus* was higher in the NC group. However, *Erysipelotrichaceae*, *Dubosiella,* and *Verrucomicrobiota* had higher relative abundance in the MC group. In addition, the relative abundance of *Lachnospiraceae*, *Clostridia* and *Lactobacillales* was higher in GAS-L, and *Staphylococcales*, *NK4A214* and *Bilophila* were higher in GAS-H (Figure 3F,G).

### 2.4. The Effects of GAS on Intestinal Metabolites in HFD/STZ Diabetic Mice

**Figure 4:**
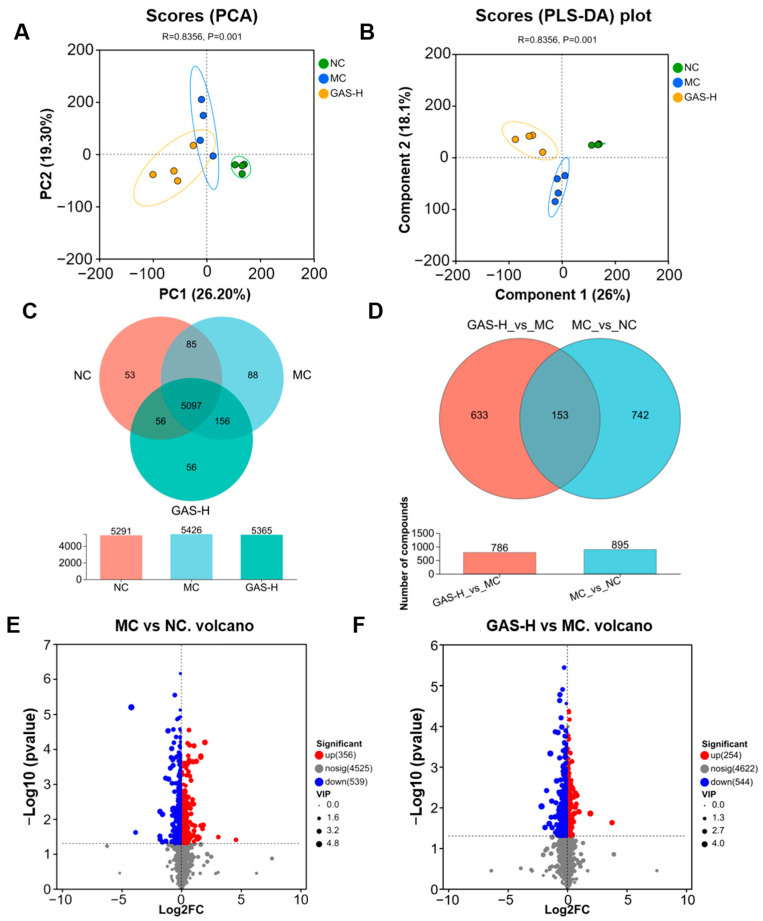
The effects of GAS on the metabolomic profiles of T2DM mice. (**A**) PCA analysis; (**B**) PLS-DA analysis; (**C**) Venn diagram; (**D**) Differential Venn diagram; (**E**) Volcano plot of the MC group vs. the NC group; (**F**) Volcano plot of the GAS-H group vs. the MC group.

Principal component (PCA) and partial least squares discriminant analysis (PLS-DA) were used to screen differential metabolites between groups, and the analysis results showed significant differences between groups with good intra-group reproducibility (Figure 4A,B). There were 5097 common metabolites among the three groups, of which 153 differentially enriched metabolites were changed in the MC group, the NC group and the GAS-H group. Finally, a total of 895 differentially abundant metabolites were identified in the MC group and the NC group, of which 353 metabolites were up-regulated and 542 metabolites were down-regulated in the MC group. A total of 786 differentially enriched metabolites were identified in the GAS-H group and the MC group, of which 235 metabolites were up-regulated and 551 metabolites were down-regulated in the GAS-H group (Figure 4C–F).

**Figure 5:**
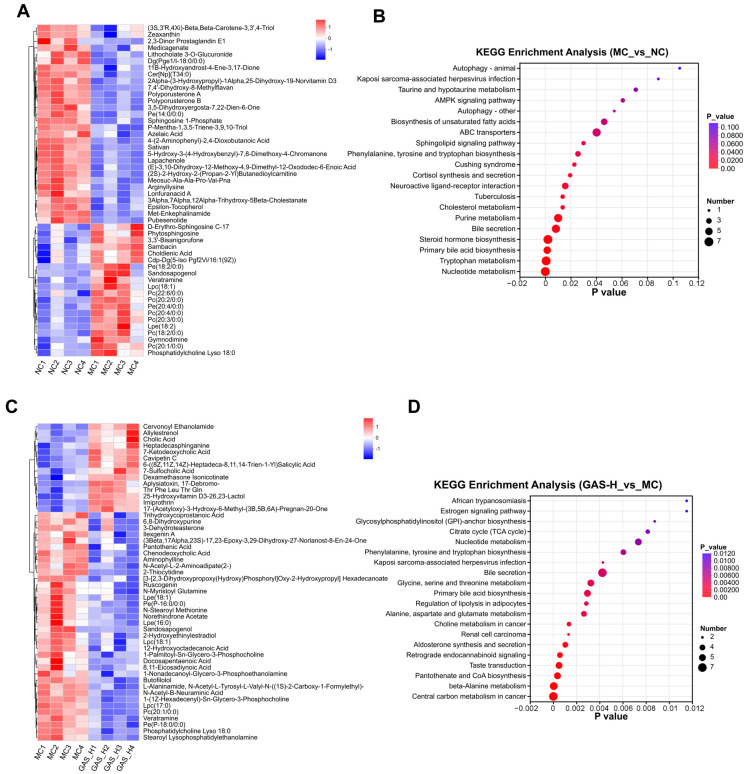
Differential metabolite screening and pathway enrichment in T2DM mice. (**A**) Heat map of the top 50 differential metabolites in the MC group vs. the NC group; (**B**) Based on KEGG pathway enrichment, a bubble map of the MC group and the NC group differential metabolites; (**C**) Heat map of the top 50 differential metabolites in the GAS-H group vs. the MC group; (**D**) Based on the GAS-H group and the MC group differential metabolites, a KEGG pathway enrichment bubble map.

The significantly altered differential metabolites were further investigated by KEGG pathway enrichment analysis to explore the potential mechanism of GAS-H in improving the symptoms of T2DM mice. The heat map results show that the MC group and the NC group shared bile secretion and primary bile acid biosynthesis pathways with the GAS-H group and the MC group. We hypothesized that GAS-H may improve disease symptoms mainly by regulating bile acid metabolism in T2DM mice (Figure 5A–D).

### 2.5. The Relationship Between Gut Microbiota and Parameters Related to T2DM

**Figure 6:**
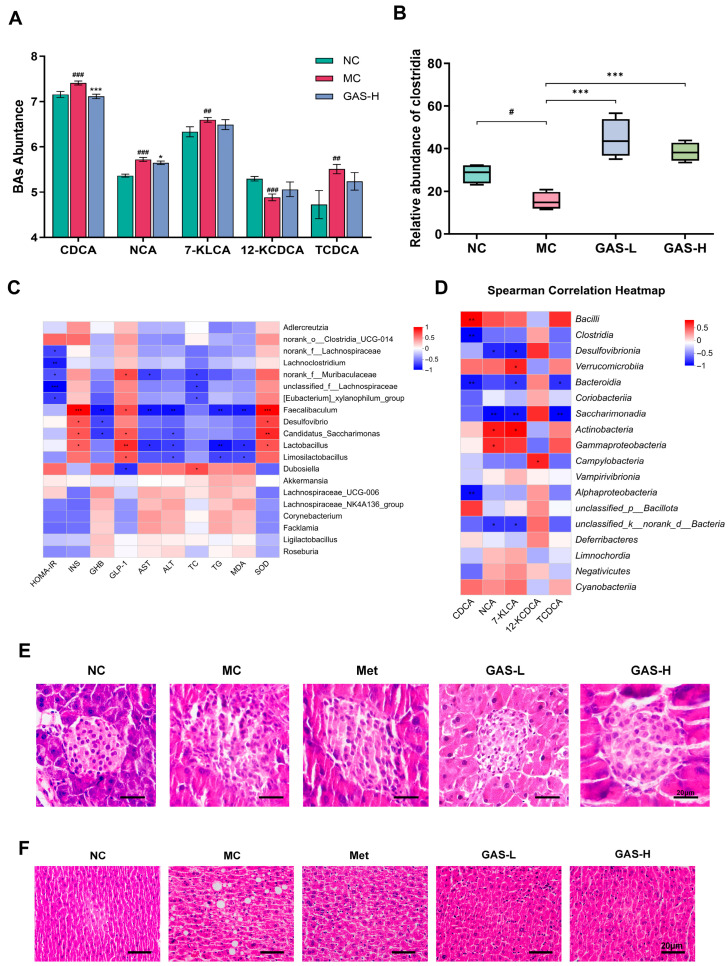
The relationship between gut microbiota and parameters related to T2DM. (**A**) Levels of bile acids in metabolites; (**B**) relative abundance of Clostridium species in metabolites; (**C**) heat map of the relationship between gut microbiota and parameters related to T2DM; (**D**) heat map of the relationship between bile acid levels and gut microbiota; (**E**) HE staining of pancreatic tissue sections from T2DM mice; and (**F**) HE staining of liver tissue sections from T2DM mice. Statistical significance: # *p* < 0.05, ## *p* < 0.01, ### *p* < 0.001, MC group vs.NC group; * *p* < 0.05, ** *p* < 0.01, *** *p* < 0.001, Treatment groups vs. MC group.

The results revealed that the levels of CDCA, NCA, 7-KLCA, and TCDCA in the MC group were significantly higher than those in the NC group, whereas 12-KCDCA was significantly lower. Following GAS-H intervention, the profiles of several bile acids shifted toward those observed in healthy mice. In the gut, primary bile acid metabolism is primarily mediated by *Clostridium* species [31]. As shown in the figure, the relative abundance of *Clostridium* species was elevated in the treated group, suggesting that pharmacological intervention enhanced the intestinal colonization of *Clostridium* species, thereby promoting the conversion of primary bile acids into secondary bile acids (Figure 6A,B).

The changes of physiological and biochemical parameters are closely related to the function of gut microbiota. To explore the key microbiota that may contribute to the beneficial effects of gastrodin, we used Spearman correlation analysis to explore the relationship between significantly altered gut microbiota at the genus level and relevant parameters. The results showed positive correlations of *Faecalibaculum*, *Lactobacillus*, *Desulfovibrio* and *Candidatus_Saccharimonas* with INS, GLP-1 and SOD, alongside negative associations with serum GHB, TG, ALT, AST and MDA. *norank_f__Lachnospiraceae*, *Lachnoclostridium*, *norank_f__Muribaculaceae*, *unclassified_f__Lachnospiraceae* and *[Eubacterium]_xylanophilum_group* were negatively associated with HOMA-IR. *Dubosiella* showed a negative association with GLP-1 and a positive association with serum TC. *Limosilactobacillus* exhibited a positive association with GLP-1, but negative associations with serum ALT, TG and MDA. (Figure 6C).

Furthermore, to determine the association between changes in BAs levels and gut microbiota, Spearman correlation analysis was performed between the five differentially expressed BAs and the top 20 classes of gut microbiota. The results revealed negative associations of *Clostridia*, *Bacteroidia* and *Alphaproteobacteria* with CDCA, as well as of *Desulfovibrionia*, *Saccharimonadia* and *unclassified_k__norank_d__Bacteria* with Nordeoxycholic acid (NCA) and 7-Ketolithocholic acid (7-KLCA). *Bacteroidia* and *Saccharimonadia* also showed negative correlations with Taurochenodeoxycholic acid (TCDCA). Conversely, *Bacilli* displayed a positive correlation with CDCA, while *Actinobacteria* and *Gammaproteobacteria* were positively associated with NCA. *Verrucomicrobiia* and *Actinobacteria* exhibited positive correlations with 7-KLCA, and *Campylobacteria* showed a positive correlation with 12-Ketochenodeoxycholic acid (12-KCDCA) (Figure 6D). Taken together, these correlations provide evidence that gut microbiota plays a crucial role in ameliorating metabolic disorders in T2DM mice (Figure 6D).

### 2.6. The Effect of GAS on Islet and Liver Morphology in HFD + STZ Diabetic Mice

The HE staining of the pancreas showed that the morphology and structure of the pancreas in the normal control group were intact; the number of islet cell clusters was sufficient and the distribution was uniform. The cells in the islets were densely arranged, with normal cell morphology, uniform cytoplasm, a clear nucleus, and no obvious inflammatory cell infiltration, edema, degeneration, or necrosis. The MC group exhibited marked pathological alterations in pancreatic tissue. Islet cell clusters were significantly reduced in both number and volume, with loosely arranged cells and widened intercellular spaces. Furthermore, numerous islet cells underwent degeneration and necrosis, accompanied by cytoplasmic vacuolization and nuclear pyknosis. The pathological damage of pancreatic tissue was obviously repaired in the GAS group, which could effectively reduce the inflammatory response of pancreatic tissue, and promote the repair of islet cells (Figure 6E).

The results of liver HE staining showed that in the normal control group, the liver tissue morphology and structure were complete, the hepatic lobules were clear in outline, the hepatocytes were arranged in order, radially distributed around the central vein, the cytoplasm of hepatocytes was uniform and transparent, the nucleus was centered and regular in shape, the nuclear membrane was intact, the nucleolus was clearly visible, and the hepatic sinusoid space was normal. There were no obvious inflammatory cell infiltration, hepatocyte edema, steatosis or necrosis. The liver tissue of the MC group showed obvious pathological damage characteristics: the structure of hepatic lobules was disordered and the boundary was unclear. The arrangement of hepatocytes was disordered, a large number of hepatocytes showed significant steatosis, and lipid droplets of different sizes were seen in the cytoplasm. Lipid droplets fused in some hepatocytes and showed macrovesicular steatosis, resulting in increased cell volume and irregular morphology. The pathological damage of liver tissue in the GAS group was significantly improved. Compared with the metformin group, the GAS group had more complete repair of hepatocyte steatosis and inflammatory cell infiltration, and the recovery of hepatic lobular structure and normal hepatocyte morphology was closer to the normal control group. This indicates that GAS has a better pathological repair efficacy in improving diabetic liver injury (Figure 6F).

### 2.7. GAS Regulates Bile Acid Metabolism Through the YY1–FXR Axis to Reduce Blood Glucose

**Figure 7:**
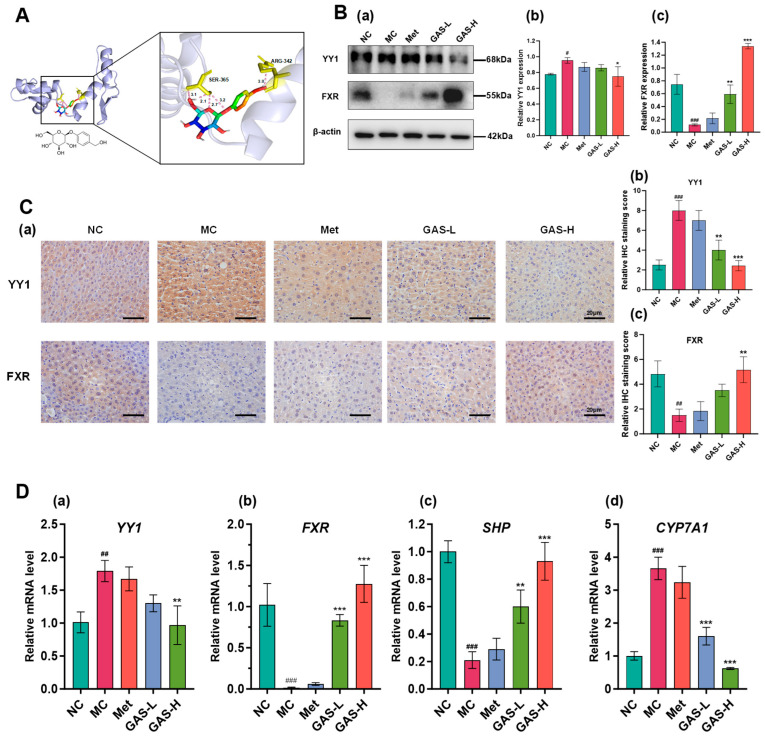
GAS regulates bile acid metabolism through the YY1–FXR axis in T2DM mice. (**A**) Molecular docking simulation of gastrodin and YY1 and interacting amino acid sites; (**B**) (**a**) Expression levels and quantitative analysis of (**b**) YY1 and (**c**) FXR in liver tissues; (**C**) (**a**) Immunohistochemical staining results of liver tissue from T2DM mice and (**b**,**c**) immunohistochemical scores of liver tissues from T2DM mice; and (**D**) The mRNA levels of (**a**) *YY1*, (**b**) *FXR*, (**c**) *SHP*, and (**d**) *CYP7A1* in liver tissues. Statistical significance: # *p* < 0.05, ## *p* < 0.01, ### *p* < 0.001, MC group vs. NC group; * *p* < 0.05, ** *p* < 0.01, *** *p* < 0.001, Treatment groups vs. MC group.

In order to further explore how gastrodin regulates blood glucose through the bile acid metabolic pathway, multiple proteins were screened. Among them, YY1 protein is involved in the regulation of many core physiological processes of the body and has been studied in a variety of diseases. Molecular docking was used to predict the binding of gastrodin to YY1. The results showed that gastrodin had two binding sites with YY1, namely ARG-342 and SER-365, the binding energy is −5.4 kcal/mol, indicating that gastrodin could bind to YY1 protein and influence its function (Figure 7A).

Western blot was used to detect the expression levels of YY1 and FXR in mouse liver tissues. As shown, in the liver tissue of T2DM mice, YY1 protein expression levels were increased due to high-fat diet and hyperglycemic disease, and its expression was down-regulated after GAS treatment (*p* < 0.05). In contrast, FXR expression was extremely low in the disease state and was significantly upregulated after GAS treatment (*p* < 0.001). This indicated a negative regulatory relationship between YY1 and FXR (Figure 7B).

In addition, the collected liver tissues were fixed in formalin fixative. After dehydration, embedding and sectioning, immunohistochemical staining was performed to detect the expression of YY1 and FXR. The results showed that compared with the control group, the expression of YY1 was increased and the expression of FXR was decreased in the MC group (*p* < 0.05), and the expression of YY1 was increased and the expression of FXR was inhibited in T2DM under the high-fat diet condition. GAS treatment decreased the expression of YY1 and increased the expression of FXR in a dose-dependent manner (*p* < 0.01). The results were consistent with those of WB (Figure 7C). Taken together, GAS treatment suppressed the expression of YY1 and promoted the expression of bile-acid-related proteins.

In order to further explore the regulatory mechanism of gastrodin in bile acid metabolism in T2DM, the liver tissues of mice were detected by qRT-PCR. *Small Heterodimer Partner (SHP)* and *CYP7A1* are both key genes in the bile acid metabolism pathway. In the bile acid metabolism pathway, *SHP*, as the core downstream transcriptional repressor of *FXR*, specifically inhibits the transcription of *CYP7A1*, the rate-limiting enzyme in the classical bile acid synthesis pathway, thereby inhibiting bile acid synthesis and maintaining bile acid homeostasis. Gastrodin inhibited the expression of *YY1* and *CYP7A1* genes and promoted the expression of *FXR* and *SHP* genes in the liver of the model group (*p* < 0.05). This indicated that after gastrodin administration, the expression level of *YY1* was decreased, which promoted the expression of *FXR* and its downstream target gene *SHP*, thereby blocking the transcription of the downstream target gene *CYP7A1* (Figure 7D). It can reduce the production of primary bile acids in the liver, improve the body’s sensitivity to insulin, and achieve the purpose of lowering blood glucose.

## 3. Discussion

Type II diabetes mellitus is a chronic metabolic disease with a high incidence in the world. Its core pathological features are insulin resistance and impaired islet-β-cell function, accompanied by glucose and lipid metabolism disorders, chronic low-grade inflammation, oxidative stress imbalance and intestinal flora imbalance [32,33]. Disorders of bile acid metabolism serve as a critical link connecting insulin resistance, gut microbiota dysbiosis, and abnormalities in glucose and lipid metabolism. Bile acids can regulate hepatic gluconeogenesis by activating signaling molecules such as the FXR receptor and the G-protein-coupled receptor TGR5, playing a central regulatory role in the pathogenesis of type 2 diabetes mellitus [34]. Studies have demonstrated that gastrodin can ameliorate complications such as diabetic encephalopathy [35] and cardiovascular diseases caused by diabetes mellitus [36]. In this study, the focus is on gastrodin’s hypoglycemic mechanism through the YY1–FXR pathway in regulating the bile-acid-metabolism pathway. The findings reveal that gastrodin can improve the core pathological phenotypes of type II diabetes mellitus through multidimensional and multi-pathway mechanisms.

At the level of glucose and lipid metabolism regulation, gastrodin enhances insulin sensitivity, reduces insulin resistance, inhibits sustained elevation of blood glucose caused by abnormal glucose metabolism, and simultaneously decreases abnormal lipid accumulation in the body. At the level of microenvironmental regulation, gastrodin mitigates inflammatory responses, strengthens antioxidant capacity, repairs cellular damage and functional disturbances induced by oxidative stress imbalance, and reduces the incidence of diabetic complications in a type II diabetes mouse model.

Disruption of the gut microbiota structure further exacerbates metabolic disorders, inflammatory responses, and insulin resistance, creating a vicious cycle of “microbiota dysbiosis–metabolic abnormalities–organ damage.” This study demonstrates that gastrodin significantly enhances the diversity of the gut microbiota in mice with type II diabetes mellitus, reduces the ratio of Firmicutes to Bacteroidetes, and increases overall gut microbial diversity. Gastrodin intervention remodels the structure of healthy gut microbiota by improving the function of the gut microecological barrier and blocking metabolic dysregulation pathways mediated by microbial dysbiosis, providing a novel microecological mechanism for how gastrodin regulates metabolic disturbances in type II diabetes mellitus.

At the molecular metabolic level, YY1, as the key binding protein of gastrodin, is the core target of regulating downstream metabolic pathways. Gastrodin binding protein YY1 negatively regulates the expression and activity of hepatic bile acid receptor FXR. FXR can promote the expression of transcriptional repressor SHP in the bile acid pathway, thereby inhibiting the activity of CYP7A1, which is related to the synthesis of primary bile acids in the liver, and reduce the abnormal synthesis and secretion of primary bile acids in the liver, thereby preventing the breakdown of unrecycled primary bile acids into secondary bile acids by intestinal microorganisms. The restoration of bile acid metabolic homeostasis can further activate intestinal endocrine function, promoting GLP-1 secretion, enhancing insulin secretion, reducing blood glucose levels, and improving symptoms of type II diabetes mellitus.

The onset and progression of diabetes mellitus are closely associated with insulin resistance, which can lead to multi-organ damage in diabetic patients. Metformin enhances insulin sensitivity but has no effect on improving pancreatic-β-cell function [21]. Unlike metformin, gastrodin exhibits reparative effects on pancreatic islet degeneration and atrophy. Furthermore, based on HE staining results of liver tissue, gastrodin alleviates hepatic pathological damage in type II diabetes mellitus mice, restores normal morphology and function of liver tissue, and breaks the vicious cycle between diabetes and liver injury.

**Figure 8:**
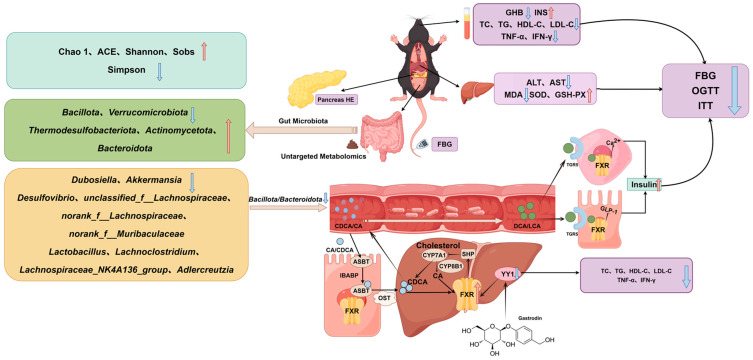
Schematic illustration of the mechanism by which gastrodin improves type II diabetes through the YY1–FXR–bile acid axis. The red upward arrow indicates that the level of this index increased after gastrodin treatment compared with the MC group. Blue downward arrows indicate a decrease in the level of this index after gastrodin treatment compared with MC group.

In summary, gastrodin not only synergistically regulates glucose and lipid metabolism, inflammatory responses, oxidative stress, and gut microbiota balance, but also provides direct pathological evidence for improving diabetes and liver complications through multiple mechanisms and targets. The specific mechanism of action is shown in Figure 8. This compound offers theoretical support for the development of precision-targeted drugs and lays an experimental foundation for novel natural hypoglycemic agents, thereby advancing the modernization and application of traditional Chinese medicine in the field of metabolic diseases.

## 4. Materials and Methods

### 4.1. Animal Experiments

Male C57BL/6J (6-week-old) mice were purchased from SPF (Beijing) Biotechnology Co., Ltd., Beijing, China. This animal research protocol has been approved by the Ethics Committee of Tianjin University of Science and Technology (Ethics Number: 2024043, 10 September 2024). All mouse experiments were conducted in strict compliance with the relevant provisions of the Ethical Guidelines for Animal Experiments. Animals were fed and watered AD libitum during rearing, and the room temperature was maintained at 20–25 °C and humidity at 40–60%. After a week of adaptation, the mice were randomly divided into the control group (NC group, *n* = 6) and the high-fat diet group (HFD group, *n* = 24). All mice had an initial body weight of 18–20 g. The HFD group was fed with a high-fat diet for 4 weeks (SPF (Beijing) Biotechnology Co., Ltd. D12079B). The high-fat diet has a total caloric density of 4.68 kcal/g and provides approximately 4 kcal/g for protein, 4 kcal/g for carbohydrate, and 9 kcal/g for fat. The protein, carbohydrate, and fat account for approximately 17%, 43%, and 41% of total calories, respectively. When the body weight reached about 30 g, the HFD mice were intraperitoneally injected with STZ (0.1 M cold citrate buffer, pH 4.5, Solarbio Science & Technology, Co., Ltd. Beijing, China) at 50 mg/kg BW for 5 consecutive days. At week 6–7, FBG was measured for three consecutive days to determine whether the type II diabetes model was successfully established. The HFD mice were randomly divided into four groups (*n* = 6), including the model group (MC), the metformin group (Met, 200 mg/kg, Merck Pharmaceutical Manufacturing, Co., Ltd. Nantong, China), the gastrodin low-dose group (GAS-L, 80 mg/kg), and the gastrodin high-dose group (GAS-H, 160 mg/kg, Aladdin Biochemical Technology, Co., Ltd., Shanghai, China). The control group and the model group were given the same amount of 0.9% NaCl by gavage every day. The HFD group was maintained on high-fat diet for 28 days. After 4 weeks of treatment, the mice were sacrificed and dissected.

### 4.2. Fasting Blood Glucose

During the experiment, we recorded the fasting blood glucose levels of mice for 12 h on the day before administration (Day 0), and on Days 4, 8, 12, 16, 20, 24, and 28 after administration. After disinfecting their tails, venous blood was collected using a blood collection needle. A drop of blood was automatically adsorbed onto a glucose test strip, and the reading from a glucometer (Roche Diagnostics (Shanghai) Co., Ltd., Shanghai, China) was awaited to determine the fasting blood glucose levels of the mice.

### 4.3. Oral Glucose Tolerance Test (OGTT) and Insulin Tolerance Test (ITT)

The oral glucose tolerance test (OGTT) and the insulin tolerance test (ITT) were performed in the last week of the experiment. Mice were fasted for 12 h and then gavaged with 20% glucose solution (2 g/kg BW). The levels of FBG in tail vein blood of mice were measured before glucose administration and at 15, 30, 60, 90 and 120 min after glucose administration. After fasting for 4 h, mice were subjected to ITT by intraperitoneal injection of 0.5 u/mL 0.5 IU/kg BW. Blood samples were collected from the tail vein of mice before and at 15, 30, 60, 90 and 120 min after administration for determination of FBG levels. The trapezoidal rule was used to calculate the area under the curve. GraphPad Prism10 was used to plot and analyze the data, and the area under the curve was calculated.

### 4.4. Serum Insulin (INS) and Glycated Hemoglobin (GHB)

In the experiment, mice were fasted for 12 h prior to euthanasia. Eye blood was collected in a 1.5 mL centrifuge tube and centrifuged at 4 °C, 3000 rpm for 10–15 min. The upper serum layer was transferred to a new EP tube, yielding approximately 150–200 μL of serum per mouse. According to the instructions of the kit, an insulin ELISA detection kit (Ruixin Biological Technology, Co., Ltd. Quanzhou, China, RXW202485M) was used to detect the insulin content in the serum. Serum total glycosylated hemoglobin was measured by a glycosylated hemoglobin ELISA kit (Ruixin Biological Technology, Co., Ltd. Quanzhou, China, RX202604M).

The detection of insulin and glycated hemoglobin includes the preparation of standard curves and sample testing. A total of 50 μL of 0, 1.25, 2.5, 5, 10, 20 and 40 mIU/L standard samples were added to each well for plotting insulin standard curves and 50 μL of 0, 12.5, 25, 50, 100, 200 and 400 ng/mL standard samples were added to each well for plotting glycated hemoglobin standard curves, where 10 μL of serum sample and 40 μL of sample diluent were added to the test well for detecting insulin or glycated hemoglobin content. After incubating at 37 °C for 15 min, the reaction was terminated. The absorbance (OD value) was measured at 450 nm using a multifunctional enzyme-linked immunosorbent assay reader (BioTek Instruments, Inc., Winooski, VT, USA. Synergy HTX). The levels of insulin or glycated hemoglobin in the samples were calculated by fitting a standard curve.

### 4.5. HOMA-IR and HOMA-β

The homeostasis assessment index of insulin resistance (HOMA-IR) and homeostasis assessment index of islet β cell (HOMA-β) were calculated by combining serum insulin content with fasting blood glucose.

$$\mathrm{HOMA-IR}=\frac{\mathrm{FBG}\;(\mathrm{mmol}/L)\;\times \;\mathrm{INS}\;(\mathrm{mU}/L)}{22.5}$$

$$\mathrm{HOMA-}\beta =\frac{20\;\times \;\mathrm{INS}\;(\mathrm{mU}/L)}{\mathrm{FBG}\;(\mathrm{mmol}/L)\;-\;3.5}$$

### 4.6. Determination of TG and TC

For TG and TC detection, blank wells, standard wells, and sample wells were set up separately. A 2.5 μL aliquot of the standard solution or serum sample was added to each well, and then 250 μL working solution was added to detect TG or TC. After incubation at 37 °C for 10 min, the OD value was read by a multifunctional enzyme-linked immunosorbent assay reader (BioTek Instruments, Inc., Winooski, VT, USA. Synergy HTX) at 500 nm. According to the TG (A110-1-1, Nanjing Jiancheng BioEngineering Co., Ltd., Nanjing, China) and TC (A111-1-1, Nanjing Jiancheng BioEngineering Co., Ltd., Nanjing, China) ELISA instruction manual, the TG and TC contents were calculated by multiplying the ratio of the OD value of the sample well to the OD value of the standard sample by the standard coefficient.

### 4.7. Determination of HDL-C and LDL-C

For HDL-C or LDL-C detection, a 2.5 μL serum sample was added to each well, and then 180 μL of working solution 1 was added and incubated at 37 °C for 5 min. The OD value was read by a multifunctional enzyme-linked immunosorbent assay reader (BioTek Instruments, Inc., Winooski, YT, USA. Synergy HTX) at 546 nm and marked as A1. Next, 60 μL of working solution 2 was added and incubated at 37 °C for 5 min; the OD value at 546 nm was record as A2. ∆A = A2 − A1. According to the HDL-C (A112-1-1, Nanjing Jiancheng BioEngineering Co., Ltd., Nanjing, China) and the LDL-C (A112-1-1, Nanjing Jiancheng BioEngineering Co., Ltd., Nanjing, China) manufacturer’s instructions, the HDL-C and LDL-C contents were calculated by multiplying the ratio of the OD value of the sample well to the OD value of the standard sample by the standard coefficient.

### 4.8. Inflammatory Factors

The serum inflammatory factors tumor necrosis factor-α (TNF-α) (Quanzhou Ruixin Biotechnology Co., Ltd., Quanzhou, China. RXW202412M) and interferon-γ (IFN-γ) (RX203097M) were detected according to the instructions of the detection kit. The detection of TNF-α and IFN-γ includes the preparation of standard curves and sample testing. A total of 50 μL of 0, 20, 40, 80, 160, 320 and 640 pg/mL standard samples were added to each well for plotting TNF-α standard curves, 50 μL of 0, 25, 50, 100, 200, 400 and 800 pg/mL standard samples were added to each well for plotting IFN-γ standard curves, and 10 μL of serum sample and 40 μL of sample diluent were added to the test well for detecting TNF-α or IFN-γ content. After incubating at 37 °C for 15 min, the reaction was terminated. The absorbance (OD value) was measured at 450 nm using a multifunctional enzyme-linked immunosorbent assay reader (BioTek Instruments, Inc., Winooski, VT, USA. Synergy HTX). The levels of TNF-α or IFN-γ in the samples were calculated by fitting a standard curve.

### 4.9. Oxidative Stress Indicators

Fresh mouse liver tissue was obtained, and tissue homogenates were prepared by pre-cooling with physiological saline. The supernatant was collected by centrifugation. Procedures were strictly followed according to the instructions of the MDA (Beyotime Biotechnology Co., Ltd., Shanghai, China, S0131) detection kit, employing the thiobarbituric acid colorimetric method, with OD values measured at 532 nm using an enzyme microplate reader (BioTek Instruments, Inc., Winooski, VT, USA. Synergy HTX). The MDA content levels in liver tissues of each group were calculated based on the standard curve and tissue protein concentration.

The supernatant of liver tissue homogenate was collected, and the total protein concentration of the samples was corrected using the BCA method. SOD (Beyotime Biotechnology Co., Ltd., Shanghai, China, S0101) activity was measured by the WST-8 assay; the reaction system was prepared according to the instructions of the SOD detection kit. After incubation under light protection at a constant temperature, the absorbance of samples was measured at 450 nm using an enzyme microplate reader (BioTek Instruments, Inc., Winooski, VT, USA. Synergy HTX). The SOD activity levels in mouse liver tissues of each group were calculated based on the inhibition rate.

The supernatant of the liver tissue homogenate was collected, and the total protein concentration of samples was corrected using the BCA method. The reaction system was prepared according to the instructions of the GSH-PX (Beyotime Biotechnology Co., Ltd., Shanghai, China, S0057S) detection kit. After incubation at a constant temperature, the OD values at 412 nm were measured using an enzyme microplate reader (BioTek Instruments, Inc., Winooski, VT, USA. Synergy HTX), and the GSH-PX activity levels in the liver tissues of each group of mice were ultimately calculated.

### 4.10. Determination of ALT and AST

Mouse liver tissues were collected and alanine aminotransferase (ALT, C009-2-1) and alanine aminotransferase (AST, C010-2-1) levels were measured according to the instructions of the assay kit (Nanjing Jiancheng Bioengineering Co., Ltd., Nanjing, China).

The supernatant from mouse liver tissue homogenate was obtained and the control and sample wells set up. A total of 5 μL of sample and 20 μL of Reagent I were added to the sample wells, while the control wells contained only an equal volume of Reagent I. After reacting at 37 °C for 30 min, 5 μL of sample and 20 μL of Reagent II were added to the control wells, and the sample wells received only an equal volume of Reagent II. After reacting at 37 °C for 20 min, 200 μL of 0.4 mol/L NaOH was added to both groups and the wells were incubated at room temperature for 15 min. The OD values were measured at 510 nm using a microplate reader and plotted against the standard curve to calculate the corresponding AST activity; the OD values were measured at 505 nm using the microplate reader and plotted against the standard curve to calculate the corresponding ALT activity.

### 4.11. Determination of GLP-1

Colonic tissue was rapidly obtained from euthanized mice, thoroughly rinsed with pre-cooled PBS, and weighed precisely. To inhibit the in vitro degradation of GLP-1 by dipeptidyl peptidase-4 (DPP-4) during sample processing, the tissue was homogenized on ice using an acidic ethanol extraction buffer (74% ethanol: 25% water: 1% 12 M hydrochloric acid, volume ratio) supplemented with Diprotin A (0.1 mM) and a broad-spectrum protease inhibitor mixture (tissue-to-buffer ratio 1:9, weight ratio). The homogenate was incubated overnight at 4 °C to ensure complete peptide extraction. Subsequently, the sample was centrifuged at 3000× *g* for 20 min at 4 °C and the supernatant collected. Detection was performed according to the ELISA kit instructions (Quanzhou Ruixin Biotechnology Co., Ltd., Fujian, China. RX202476M). The detection of GLP-1 includes the preparation of standard curves and sample testing. A total of 50 μL of 0, 37.5, 75, 150, 300, 600 and 1200 pg/mL standard samples were added to each well for plotting GLP-1 standard curves. After incubating at 37 °C for 15 min, the reaction was terminated. The absorbance (OD value) was measured at 450 nm using a multifunctional enzyme-linked immunosorbent assay reader (BioTek Instruments, Inc., Winooski, VT, USA. Synergy HTX). The levels of GLP-1 in the samples were calculated by fitting a standard curve. All GLP-1 values were standardized by total protein content, which was determined by BCA assay.

### 4.12. Western Blot Analysis

Liver tissue was washed with PBS and then RIPA strong lysate (Beyotime Biotechnology, Shanghai, China) and a protease inhibitor cocktail (MedChemexpress, Monmouth Junction, NJ, USA) was added. The samples were ground and lysed on ice. The lysate was centrifuged at 12,000 rpm for 10 min and the supernatant was removed. The BCA Protein Assay Kit (Beyotime Biotechnology, Shanghai, China) was employed to ascertain the protein concentration. To separate the total protein, SDS-PAGE was utilized, and then the proteins were transferred onto a PVDF membrane (Merckmillipore, Darmstadt, Germany). This membrane was then subjected to blocking with 5% skim milk for 1 h. Subsequently, it was incubated overnight at 4 °C with the following primary antibodies: YY1 (Proteintech, 1:20,000) and FXR (Abmart, 1:1500). After the incubation period, the membrane underwent three 10 min washes with TBST at room temperature. Subsequently, it was incubated for an hour at room temperature with secondary antibody (YEASEN, Shanghai, China, 1:500). The strips were visualized using a SuperKine™ ECL (Abbkine Biotechnology Co., Ltd., Atlanta, GA, USA) along with a Chemiluminescent Imaging System (Image Quant LAS 4000, General Electric, Boston, MA, USA).

### 4.13. Hematoxylin-Eosin Staining

Liver and pancreas samples were fixed, dehydrated, and embedded in paraffin. Paraffin-embedded tissues were made into 5 µm sections. Sections were deparaffinized and rehydrated before staining. Hematoxylin-eosin (HE) staining was performed using standard methods. The cytoplasm was stained with eosin (ZSGB-BIO, Beijing, China), and the nucleus was stained with hematoxylin (ZSGB-BIO, Beijing, China). All tissue sections were examined under a light microscope (Olympus, Tokyo, Japan).

### 4.14. Immunohistochemistry

Mouse liver tissue samples were fixed in 4% paraformaldehyde, embedded in dehydrated paraffin and serially sectioned. The sections were deparaffinized, hydrated, antigen repaired and blocked with endogenous peroxidase. After blocking non-specific antigen at room temperature, the sections were incubated with primary antibodies YY1 (Proteintech, 1:200) and FXR (Abmart, 1:500) at 4 °C overnight. The next day, the plates were incubated with secondary antibody (Suzhou Immunoway Biotechnology Co., Ltd. Suzhou China. RS0011) at room temperature, stained with DAB (Beijing Solarbio Co., Ltd. Beijing China. DA1016) counterstained with hematoxylin (Beijing Solarbio Co., Ltd. Beijing China. G1080), dehydrated and transparent by gradient, and sealed. The positive expression levels and distribution characteristics of YY1 and FXR proteins in each group were analyzed.

### 4.15. 16S rRNA Sequencing

Colonic tissues were collected after the mice were sacrificed, and the colonic contents were removed and immediately transferred to 1.5 mL EP tubes. Fresh colonic contents were used for 16S rRNA gene sequencing and untargeted metabolomics analysis. The total genomic DNA of microbial communities was extracted. Purified amplicons were pooled in equimolar amounts and paired-end sequenced on an Illumina Nextseq2000 platform (Illumina, San Diego, CA, USA), according to the standard protocols by Majorbio Bio-Pharm Technology Co., Ltd. (Shanghai, China). All data are available through the Majorbio cloud platform (https://cloud.majorbio.com, accessed on 11 January 2026) on intestinal flora of bioinformatics analysis.

### 4.16. Metabolomic Analysis

Colonic contents for metabolite extraction and untargeted metabolite profiling analysis was performed via ultrahigh-performance liquid chromatography tandem mass spectrometry (UHPLC-MS/MS) by Majorbio, Inc. (Shanghai, China). The instrument platform for LC/MS analysis was a UHPLC–Q Exactive system (Thermo Fisher Scientific Inc, Waltham, MA, USA). After mass spectrometry detection was completed, the raw LC/MS data were preprocessed via Progenesis QI version 3.0 (Waters Corporation, Milford, CT, USA) software for the identification of metabolites. Further analyses were performed on the Majorbio cloud platform (https://cloud.majorbio.com, accessed on 11 January 2026). Differentially abundant metabolites between various groups were summarized and mapped to their respective biochemical pathways through metabolic enrichment and pathway analyses based on database searches (KEGG, http://www.genome.jp/kegg/, accessed on 11 January 2026).

### 4.17. Molecular Docking

The 3D structure of human YY1 was downloaded from the PDB database (PDB code 1UBD). The 3D structure of gastrodin was downloaded as an SDF file from the PubChem database and converted using Chem 3D. The YY1 protein was preprocessed using AutoDock 4. 2. 6 software to remove water molecules, add polar hydrogen atoms, and calculate Gasteiger charges; simultaneously, structural optimization and flexible bond assignments were performed for the small molecule gastrodin. The active pocket region of YY1 protein was selected, the docking grid parameters were configured, and the molecular docking was performed. Based on binding energy, hydrogen bonding interactions, and key amino acid residue binding sites, the optimal docking conformation was screened for. Finally, the docking results were visualized and enhanced with PyMOL 1. 7. 4.

### 4.18. Quantitative Reverse Transcription Polymerase Chain Reaction (qRT-PCR)

Total RNA was extracted from liver tissue using the RNA simple total RNA extraction kit (DP419, Tiangen Biotech (Beijing) Co., LTD. Beijing, China.) following the manufacturer’s instructions. The purity of the isolated total RNA was verified using an ultraviolet spectrophotometer (Shimadzu Corporation, Kyoto, Japan), with an A260/A280 ratio ranging from 1.8 to 2.0 indicating compliance with experimental standards, after which reverse transcription was performed. Reverse transcription was conducted using the Tiingen reverse transcription kit (KR1116, Tiangen Biotech (Beijing) Co., LTD. Beijing, China.): 2 μg of RNA was combined with 2 μL gDNA Buffer, adjusted to 10 μL with nuclease-free water, mixed briefly, centrifuged, incubated at 42 °C for 3 min, and placed on ice. A reverse transcription pre-mix was then prepared as instructed, added to the gDNA-removed reaction solution, mixed thoroughly, incubated at 42 °C for 15 min and 95 °C for 3 min, and chilled on ice; the resulting cDNA was stored at −80 °C. Real-time fluorescence quantitative PCR (BIO-RAD, Singapore, CFX connect) was subsequently performed using β-actin as the reference gene to determine the mRNA expression levels of *YY1*, *FXR*, *CYP7A1*, and *SHP* in mouse liver tissues.

### 4.19. Statistical Analysis

GraphPad Prism 10.0 software was used for data drawing and analysis. Paired t-test was used for comparison of two independent samples, and one-way ANOVA was used for comparison of multiple groups of data. Two-way ANOVA was used to compare multiple sets of data with two or more variants. For each experiment, data are presented as mean ± SD. Statistical significance: # *p* < 0.05, ## *p* < 0.01, ### *p* < 0.001, MC group vs. NC group; * *p* < 0.05, ** *p* < 0.01, *** *p* < 0.001, Treatment groups vs. MC group.

## 5. Conclusions

This study investigated the effects and molecular mechanisms of gastrodin in ameliorating type II diabetes mellitus. The results demonstrated that gastrodin effectively improved insulin resistance, glucose and lipid metabolism disorders, inflammatory and oxidative stress injuries in diabetic mice, restored intestinal microbiota homeostasis, and reduced the incidence of complications. Mechanistically, gastrodin targets YY1 to negatively regulate hepatic FXR, thereby modulating bile acid metabolism and promoting GLP-1 secretion to improve glucose metabolism. Additionally, gastrodin repairs damaged liver and pancreatic tissues, alleviating tissue injury caused by diabetes. These findings demonstrate gastrodin’s multifaceted therapeutic advantages, providing direct experimental evidence for its use as a replacement or adjunct in clinical glucose-lowering therapy and expanding the application prospects of natural drugs in diabetes management. However, further screening and research are required to identify other target proteins involved in gastrodin’s regulation of bile acid metabolism through the hepatobiliary axis. Furthermore, gastrodin can be combined with other drugs for in vivo and in vitro experiments to elucidate its underlying mechanisms in improving bile acid metabolism and managing type II diabetes.

## Data Availability

The original contributions presented in the study are included in the article, further inquiries can be directed to the corresponding author.
